# Lipotropes Protect against Pathogen-Aggravated Stress and Mortality in Low Dose Pesticide-Exposed Fish

**DOI:** 10.1371/journal.pone.0093499

**Published:** 2014-04-01

**Authors:** Neeraj Kumar, Subodh Gupta, Nitish Kumar Chandan, Md. Aklakur, Asim Kumar Pal, Sanjay Balkrishna Jadhao

**Affiliations:** Department of Fish Nutrition, Biochemistry and Physiology, Central Institute of Fisheries Education, Versova, Mumbai, Maharashtra, India; NIEHS/NIH, United States of America

## Abstract

The decline of freshwater fish biodiversity corroborates the trends of unsustainable pesticide usage and increase of disease incidence in the last few decades. Little is known about the role of nonlethal exposure to pesticide, which is not uncommon, and concurrent infection of opportunistic pathogens in species decline. Moreover, preventative measures based on current knowledge of stress biology and an emerging role for epigenetic (especially methylation) dysregulation in toxicity in fish are lacking. We herein report the protective role of lipotropes/methyl donors (like choline, betaine and lecithin) in eliciting primary (endocrine), secondary (cellular and hemato-immunological and histoarchitectural changes) and tertiary (whole animal) stress responses including mortality (50%) in pesticide-exposed (nonlethal dose) and pathogen-challenged fish. The relative survival with betaine and lecithin was 10 and 20 percent higher. This proof of cause-and-effect relation and physiological basis under simulated controlled conditions indicate that sustained stress even due to nonlethal exposure to single pollutant enhances pathogenic infectivity in already nutritionally-stressed fish, which may be a driver for freshwater aquatic species decline in nature. Dietary lipotropes can be used as one of the tools in resurrecting the aquatic species decline.

## Introduction

Freshwater species decline and endangerment around the world has been receiving increased attention, but there are missing spots in linking these patterns to physiological mechanisms and in finding possible remedies. It is a well-recognized multifactorial problem related to habitat destruction, climate change, and pesticide use, among which, little is known about the latter. Unsustainable trends in the use of synthetic fertilizers and pesticides are evident from their respective seven-fold and three-fold increases in last four decades [1]; application of pesticide is expected to increase by 170 percent by 2050 [2]. These trends' negative effects on ecosystem health are corroborated by an increased outbreak of diseases and greater loss of biodiversity. Among the various ecosystems being radically altered, freshwater ecosystems are the most endangered by these anthropogenic activities, because unlike in marine systems, the likelihood of pollutant dilution is rare in freshwaters systems, resulting in the suppression of fish immune systems and increase in mortality. Some 34 percent of fish species, mostly from fresh water, are threatened with extinction [3] and the number of threatened fish species in the Red List [4] (version 2011.2) has increased from 734 in 1996 to 2028 in 2011, and the list is growing every year. In India, out of a total 46 percent evaluated from 700 total freshwater fish species, 70 percent are threatened [5]. Of anthropogenic stressors, pesticides are of prime concern as they aggravate the effects of other stressors, which has negative implications for biodiversity and the aquaculture industry. In the United States, pesticides were found to pollute every stream and over 90 percent of wells sampled in a study by the US Geological Survey [6] and the situation around the world is similar.

This is an enormous challenge, and effective tools are necessary for the successful conservation of threatened species, as well as for producing an extra 37 million tons of fish and aquatic food by 2030 in order to feed the world's burgeoning population under even mildly polluted aquatic systems. From simple aquaculture producers' utilitarian perspectives, the cost of cleaning up pollution when resources are scarce could favor secondary prevention strategies, such as nutritional strategies, for mitigating this environmental insult and restoring endangered species. Nutritional strategies are handy and appropriate for combating various stressors. Available evidence indicates that stress alters metabolism, causes hypo/demethylation of DNA [7] and also changes requirement of variety of nutrients [8], [9]. Methyl groups are of vital importance as animals cannot synthesize them and thus need to receive them through diet [10]. Despite the established link between stress and methylation, direct studies on methyl donor (lipotropic compound) supplementation in an ecological context are scarce.

Although pesticide regulations are designed to protect human and wildlife communities from large-dose exposures to pesticides and prevent acute disease symptoms and mortality, nothing exists in regulations to prevent low-level exposures and sublethal effects [11], which should be a concern. Even sublethal/nonlethal environmental pollutants and pesticides like endosulfan (organochlorine lipophilic insecticide) and others at ecologically relevant doses cause immunosuppressive effects in several species [12], [13] including fish [14], affect recruitment (and reproduction), increase pathogen virulence [15] and delay development, decrease longevity, decrease foraging success and cause decline in species populations [16]. While acute toxicity aspects have been extensively investigated and there are a few discrete studies on sublethal toxicity effects in fish on cortisol secretion and glutathione-s-transferase [17] and immunological parameters [14], [18] along with an indication of increased susceptibility of juvenile Chinook salmon to vibriosis after exposure to chlorinated and aromatic compounds found in contaminated urban estuaries [19], comprehensive studies elucidating the physiological mechanisms of the nonlethal toxicity and concurrent pathogenic infections under experimental conditions along with counteractive measures are rarely attempted. This study was done in *Labeo rohita*, a commercially important freshwater carp species in the Indian subcontinent [20] contributing 80–90 percent of the carp polyculture, and almost a dozen species of this genus are endangered/threatened [4]. These experiments were carried out with the aim of studying the effects of low dose endosulfan exposure on comprehensive stress responses, including mortality in pesticide-exposed (nonlethal dose) and pathogen-challenged fish, and whether lipotropes can counteract these responses elicited by exposure of fish to pesticide.

## Material and Methods

### Ethics Statement

The use of animals conforms to the existing laws in India. The care and treatment of animals used in this study were in accordance with the guidelines of the CPCSEA [(Committee for the Purpose of Control and Supervision of Experiments on Animals), Ministry of Environment & Forests (Animal Welfare Division), Government of India] on the care and use of animals in scientific research. The study protocol and experimental endpoints were approved by the Advisory Committee of this research work, Board of Studies and authorities of the Central Institute of Fisheries Education (Deemed University), Mumbai (India). As the experimental fish *L. rohita* is a commercially important and non-endangered fish, the provisions of the Government of India's Wildlife Protection Act of 1972 are not applicable for experiments on this fish.

### Fish and Experimental Design

Fingerlings of *Labeo rohita* (average weight 7.95±0.04 g) were procured from Prem Fisheries Consultancy, Gujarat, India, and transported to the experimental facilities at the Institute in a circular container (500 L) with sufficient aeration. The animals were acclimatized to the experimental rearing conditions for 15 days. Fifteen fish of uniform size (average weight 7.96±0.04 g) per container were stocked in five distinct groups with three replicates for each treatment in plastic containers of 150 L capacity (80×57×42 cm) each following a completely randomized design. Control fish were reared in normal water and fed basal (control) feed. Fish in the four experimental groups were exposed to low dose endosulfan (1/10th dose of LC_50_) for 37 days and fed with control feed or given feed supplemented with 0.1 percent choline, or 0.5 percent betaine, or 2 percent lecithin twice daily (09:00 and 17:00 h) to approximate satiation. The LC50 of endosulfan used in this study was based on our study in same species [21]. The quality and preparation details of endosulfan have been described earlier [21], [22]. Growth was monitored on the 17th and 37th day by collectively weighing each group of fish. Fishes were starved overnight before taking the weight. Round-the-clock aeration was provided to all the containers from a compressed air pump and manual water exchange (two third) was carried out daily. Water quality parameters were checked every week using the methods of APHA [23] and the water quality conformed to carp-rearing standards (**Table S1**). As the residue determination required large quantity of fish mass, another set of fishes (average weight 262 g) procured from the same source were used in a setup similar to that given above for th same duration. This additional set allowed determination of serum caspase, HSP70, methyl transferase (MT) and vitellogenin in these fishes along with endosulfan residue in muscle tissue.

### Experimental Diets and Proximate Analysis

Four isoproteinous (35% crude protein) and isocaloric (410 kcal/100 g) practical diets were formulated: control basal feed, and basal feed containing: choline as choline chloride (SD Fine Chemicals Ltd, Mumbai) (0.1 g/kg); betaine as betaine hydrochloride (HIMEDIA, Mumbai, India) (0.5 g/kg); and lecithin (20 g/kg) as soylecithin (HIMEDIA, Mumbai, India), respectively. Choline and betaine were included at the expense of wheat flour, while lecithin (being lipid) was included at the expense of oil components in the diet. The composition of the basal diet is given in **Table 1**. Betaine and choline chloride were first dissolved in water and incorporated into a vitamin mineral premix, whereas lecithin was mixed in oil. For formulation of the pelleted diet, a manually prepared vitamin and mineral mixture along with ascorbyl phosphate (SRL Ltd., Mumbai, India) as the source of vitamin C was used. The dough was mixed properly and was pelleted, air dried and kept in a hot air oven at 60°C until dry and was subsequently stored at 4°C until required for feeding. The proximate composition of all the experimental diets and fish were analyzed as per the methods of AOAC [24].

**Table 1:** Composition of the basal diet.

Ingredients	Percent
Soybean meal^a^	45.5
Fish meal^a^	10.00
Sunflower meal^a^	10.00
Wheat flour^a^	14.97
Wheat bran^a^	10.00
Sunflower oil^a^	4.00
Cod liver oil^a^	2.00
CMC^b^ (Carboxyl methylcellulose)	1.00
Vitamin + mineral mix^c^	2.00
Vitamin C^d^	0.030
Chromic oxide	0.50

a, b, c, d Sources: ^a^procured from local market, ^b^HiMedia (JTJ Enterprises, Mumbai, India), ^c^Prepared manually and all components from HiMedia Ltd and ^d^SD Fine-Chemicals Ltd (Mumbai, India).

c Composition of vitamin mineral mix (quantity/250 g starch powder): Vitamin A 550,000 IU; Vitamin D_3_ 110,000 IU; Vitamin B_1_ 20 mg; Vitamin B_2_ 200 mg; Vitamin E 75 mg; Vitamin K 100 mg; Vitamin B_12_ 0.6 μg; Calcium Pantothenate 250 mg; Nicotinamide 1000 mg; Pyridoxine 100 mg; Mn 2,700 mg; I 100 mg; Fe 750 mg; Zn 500 mg; Cu 200 mg; Co 45 mg; Ca 50 g; P 30 g; Selenium 5 ppm.

### Sampling

At the end of the of 37-day feeding trial, the first sampling was carried out for analysis of the different blood parameters, respiratory burst and serum lysozyme activity. Three fish from each replicate with a total of nine fish from each treatment were anaesthetized with clove oil (50 μl/1) and blood was collected from the caudal vein. For serum, another six fish from each treatment were anaesthetized and blood was collected without anticoagulant and allowed to clot for 2 h followed by collection of serum with a micropipette and stored at −20°C until use. The procedures used for blood collection, estimation of blood glucose, haemogobin, total erythrocyte and total leucocyte count in Neubauer's chamber, total serum protein, albumin and globulin, the respiratory burst activity of phagocytes (as measured by intracellular superoxide radical-induced reduction of nitro blue tetrazolium (NBT), tissue homogenate preparation and analysis of enzyme (superoxide dismutase (SOD) (E.C.1.15.1.1), catalase (E.C.1.11.1.6) and glutathione-s-transferase (EC 2.5.1.18) are described earlier [22]. Serum cortisol was determined via radioimmunoassay as described [25]. For removal of organs, the required numbers of fish were anesthetized with an overdose of clove oil and cessation of heartbeat was observed.

### Quantification of Markers of Stress and Endocrine Disruption and Sex Steroids

The expression of HSP-70 (EIA kit, catalog o. EKS-700B), and caspase-3 (colorimetric detection kit, catalog no. ADI-907-013) in the gill and liver samples and serum methyl transferase (MT) (MT detection kit, catalog no. ADI-907-025) were determined as per the manufacturer's instructions (Bioguenix/Enzo Life Science, Mumbai, India). Yolk precursor protein, vitellogenin and 11-keto testosterone (11-KT) were quantified in the male and female serum using ELISA with a vitellogenin EIA kit (Catalog No. V01003402) (Biosense, Bergen, Norway) and an EIA kit (Catalog no. 582751) (Cayman Chemical Co. Ann Arbor, MI), respectively, as per the manufacturer's instructions. The absorbance was read in an ELISA plate reader (Biotek India Pvt. Ltd, Mumbai, India).

### Serum Lysozyme Activity

Serum samples were diluted with phosphate buffer (pH 7.4) to a final concentration of 0.33 mg ml^−1^. In a cuvette, 3 ml of *Micrococcus luteus* (Bangalore Genei, India) suspension in phosphate buffer (A_450_ = 0.5–0.7) and 50 μl of diluted serum sample were mixed well for 15 sec, and the reading was taken in a spectrophotometer at 450 nm exactly after 60 sec of addition of serum sample. This absorbance was compared with a standard lysozyme (Bangalore Genei, India) of known activity following the same procedure as above. The activity was expressed as U min^−1^ mg^−1^ protein of serum.

### Post-challenge Protection and Agglutinating Antibody Titre

A compound is said to have immunostimulatory properties if it meets the classical definition of an immunostimulant, which requires the administered compound to boost animal immune response to a level at which they pass a survival test following a challenge with a pathogenic microorganism. Thus, to determine if the lipotropic compounds used in the experiment meet the definition of immunostimulant in fish, a challenge test was carried out using the pathogenic bacteria *Aeromonas hydrophila*, which was cultured and prepared as described [25]. After 37 days of feeding, 24 fish per group were challenged with an intraperitoneal injection of *A. hydrophila*, and survival was monitored over a seven-day period. As it was logistically difficult to watch the challenged fish 24 hrs a day for seven days, periodic observations were carried out at a minimum interval of every six hours, and more often during the mornings. At these times dead fish were noticed more often than severely morbid (about to die) fish. As survival of fish over a period of seven days was the experimental endpoint for a pathogen challenge test, every attempt was made to keep good hygiene in the experimental tub, and any dead fish were removed as soon as noticed. Severely morbid fish, when discovered, were anaesthesized with clove oil (50 μl/1) and blood was withdrawn from the caudal vein followed by overdosing with clove oil until cessation of heartbeat was observed, and the organs were harvested to provide a better histology picture than already-dead fish could provide. At the end of the challenge, surviving fish from each group were anaesthetized, and sera samples were collected for antibody titre determination by agglutination assay as per Plumb and Areechon [26]. To prevent the spread of infection, after the end of each procedure, surviving fish were overdosed with clove oil and the cessation of heart beat was monitored.

### Determination of Bioaccumulation of Pesticide

For pesticide extraction, fish muscle (250 g) was ground in a high speed blender with excess anhydrous sodium sulfate (100 g) [24]. The lipid fraction was extracted using petroleum ether, passed through an anhydrous sodium sulfate column, and filtered. The elute was made up to a known volume. Acetonitrile and saturated petroleum ether were used for pesticide fractionation. Petroleum ether was then collected and evaporated to 10 mL. The concentrate (2 μL) was injected into GC (Shimazdu 14 B) using a capillary column of 1.85- m length, 4 mm internal diameter, made of glass, packed with 10% D.C. 200 (w/w) on solid support 80- mesh chromatosorb WHP, and measured with an electron capture detector (63 Ni). Nitrogen was the carrier gas (flow rate 30 mL/min). The column temperature was increased from 170–240°C at the rate of 10°C/min. The temperature of the detector and injector was 270°C. Residue quantification was done using appropriate standards.

### Histology

For histopathological studies, immediately after fish dissection, liver tissue was stored in 10% neutral buffered formalin (Na_2_HPO_4_: 0.6 g, NaH_2_P0_4_: 4 g, distilled water: 100 ml and formalin: 10 ml). The samples were processed and embedded in paraffin, and after blocking and cooling, sectioning (5 μm) was done using a rotatory microtome. Mounted sections were dewaxed in xylene and dehydrated serially in alcohol after embedding in paraffin wax, cut into, and stained by Haematoxylin and Eosin (H&E) as described by Roberts [27] and examined under a light microscope (Olympus CX-31, Japan).

### Statistical Analysis

The main effect among five different groups was analyzed by one way ANOVA. The comparison of any two mean values was done by Duncan's multiple range test (DMRT). The mean values for pre- and post-challenge attributes were compared by Student's t-test. The statistical analysis was performed using SPSS (version 16).

## Results

### Nonlethal Low Dose Endosulfan Exposure Elicits Primary Stress Response in Fish but Lipotropes Counteract It

**Figure 1:**
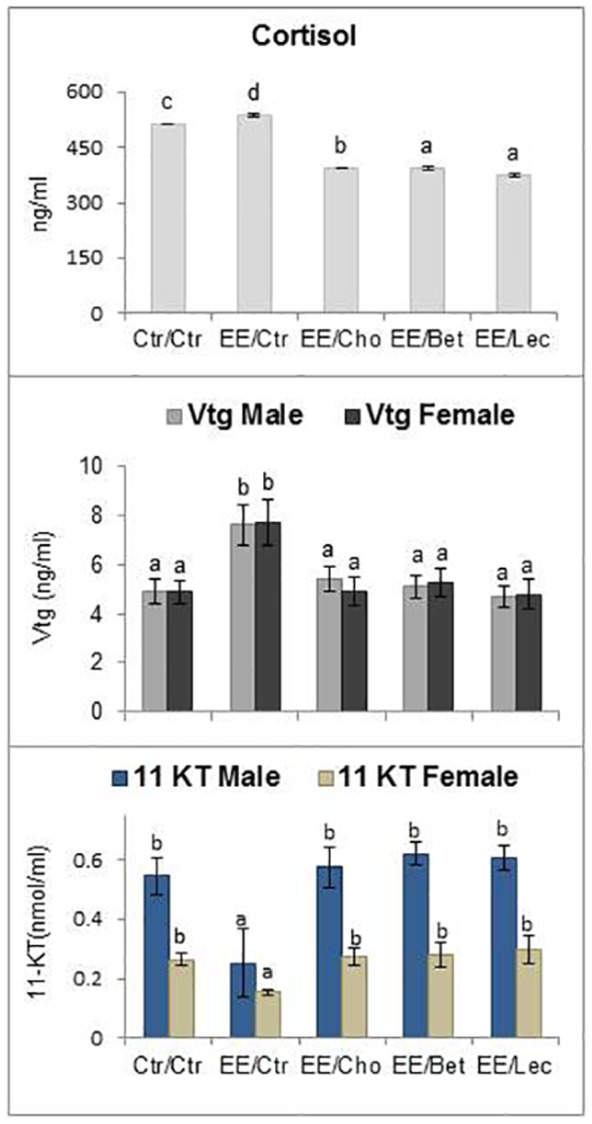
Primary stress response to low (nonlethal) dose endosulfan exposure in fish unfed or fed with lipotropes for 37 days: Steroid hormones in serum. Abbreviations for exposure/diet treatments of fish: Ctr/Ctr, fish group reared in normal/control (Ctr) water and fed control (Ctr) feed; EE/Ctr, low dose endosulfan-exposed (EE) and control feed (Ctr) fed group; EE/Cho, low dose endosulfan-exposed and supplemental choline (Cho) fed group; EE/Bet, low dose endosulfan-exposed and supplemental betaine (Bet) fed group; EE/Lec, low dose endosulfan-exposed and supplemental lecithin (Lec) fed group. The values reported in bar charts represent the mean±SE. Bars bearing different letters (a, b, c, d) indicate significant differences between treatment means for that parameter and for that particular sex. Probability (P) values: cortisol (P = 0.001), vitellogenin in males (P = 0.013), vitellogenin in females (P = 0.030), 11-Ketotestosterone (KT) in males (P = 0.02) and 11-KT in females (P* = *0.01). Number of observations (n): n = 6 for cortisol and n = 7 for vitellogenin and for 11-KT in males and females. Levels of 11-KT in females were lower (P<0.05) by student's t-test than for males in respective groups.

Primary stress response is the immediate effect on endocrine hormones following extraneous stressors. Primary stress responses in fish were quantified by measuring serum markers of stress (cortisol) and endocrine disruption (vitellogenin induction) and the male sex steroid hormone 11- keto-testosterone (11-KT). The level of serum cortisol (P<0.01) was significantly increased by exposure to endosulfan, as was the vitellogenin. Exposure decreased the level of 11-KT in the animals. Dietary lecithin, betaine and choline supplementation prevented the effects of endosulfan on these hormones from being invoked and while serum levels of cortisol were lower, those of 11-KT and vitellogenin were on par with control (**Figure 1**).

### Nonlethal Low Dose Endosulfan Exposure Elicits Cellular (Secondary) Stress Responses in Fish but Lipotropes Counteract Them

**Figure 2:**
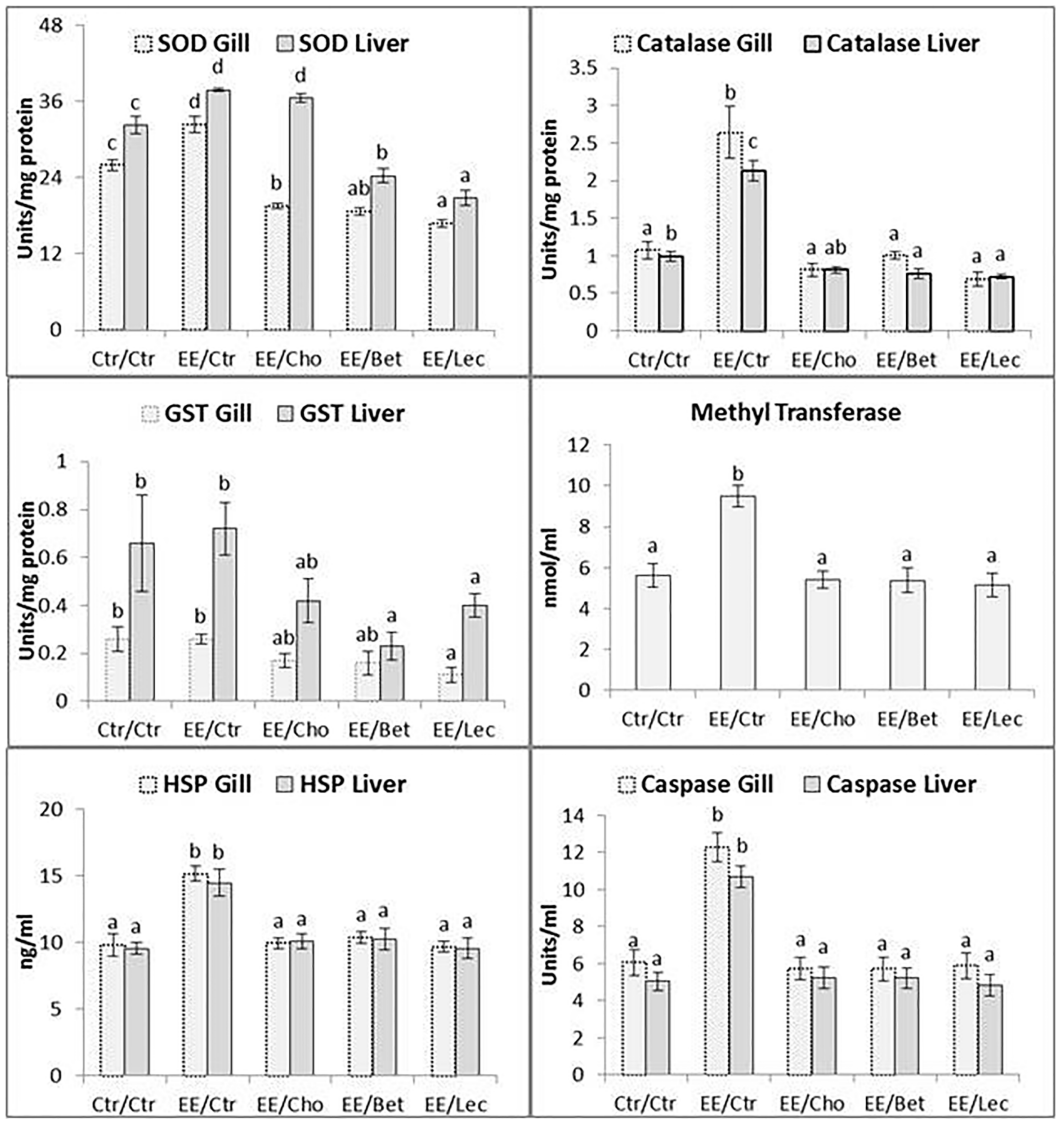
Secondary stress response to low dose endosulfan exposure in fish unfed or fed with lipotropes for 37 days: Cellular responses. Secondary cellular stress responses included activities/levels of: antioxidant enzymes superoxide dismutase (SOD) and catalase; phase II metabolism enzymes glutathione-s-transferase (GST) and SAM-dependent methyl transferase (MT); heat shock protein (HSP70); and caspase. While MT was measured in serum, all other attributes were quantified in the liver and gills. Abbreviations for exposure/diet treatments of fish are the same as used in Figure 1: Ctr, control; EE, endosulfan-exposed; Cho, choline; Bet, betaine and Lec, lecithin. The values reported in bar charts represent the mean±SE. Bars bearing different letters (a, b, c) indicate significant differences between treatment means for the level/activity of a marker in respective tissue or serum. Probability (P) values: SOD liver (P = 0.002), SOD gill (P = 0.005), catalase gill (P = 0.003), catalase liver (P = 0.005), GST gill (P = 0.02), GST liver (P = 0.03), serum MT (P* = *0.001), HSP70 and caspase (P = 0.001). Number of observations (n): n = 6 for SOD, GST, catalase, HSP70 and caspase, and n = 7 for MT.

Metabolic stress responses in terms of blood glucose, liver glycogen and body protein content were not influenced (P>0.05) by exposure to low dose endosulfan and by lecithin, betaine and choline supplementation in exposed fish (**Table 2**). However, nonlethal exposure to low dose endosulfan significantly (P<0.01) enhanced cellular stress indicators like antioxidant enzymes (superoxide dismutase and catalase), phase II enzymes in xenobiotic metabolism (glutathione-s-transferase, GST, and methyl transferase, MT), protein such as caspase-3 involved in apoptosis and heat shock proteins in gill and liver tissue. While choline, betaine and lecithin supplementations were able to prevent the effects of endosulfan (values either comparable or lower than control) on these parameters, the effects of betaine and especially lecithin were more pronounced leading to lowered values of SOD, catalase and GST in liver and gill tissue (**Figure 2**).

**Table 2:** Secondary stress response to low dose endosulfan-exposure in *L. rohita* fish fingerlings unfed or fed with lipotropes for 37 days: Effect on body composition and some metabolites.

Exposure/Diet	Control/Control	Endosulfan/Control	Endosulfan/Choline	Endosulfan/Betaine	Endosulfan/Lecithin	P-Value
Body OM^1^	86.57±0.41	85.74±0.46	84.62±1.58	86.46±0.10	86.46±0.21	0.41
Body CP^2^	64.42±3.57	58.11±0.68	61.42±6.56	59.52±1.28	60.63±4.94	0.84
Body ash^3^	13.43±0.41	15.38±1.58	13.54±0.10	13.73±0.21	14.26±0.46	0.32
Liver glycogen	0.01±0.003	0.03±0.006	0.01±0.0016	0.10±0.078	0.02±0.006	0.24
Blood glucose (mg/dL)	70.03±4.28	76.89±4.93	67.35±2.29	67.76±6.28	65.77±2.4	0.27

OM^1^, Organic Matter, CP^2^, Crude Protein and ^3^Ash expressed as % DM.

Glycogen expressed as mg glycogen/g tissue. Data expressed as Mean ± SE (n = 6).

### Pathogen Infection Aggravates Low Dose Endosulfan Exposure-induced Secondary Stress (Hemato-immunological and Histological) Responses but Lipotropes Counteract Them

**Figure 3:**
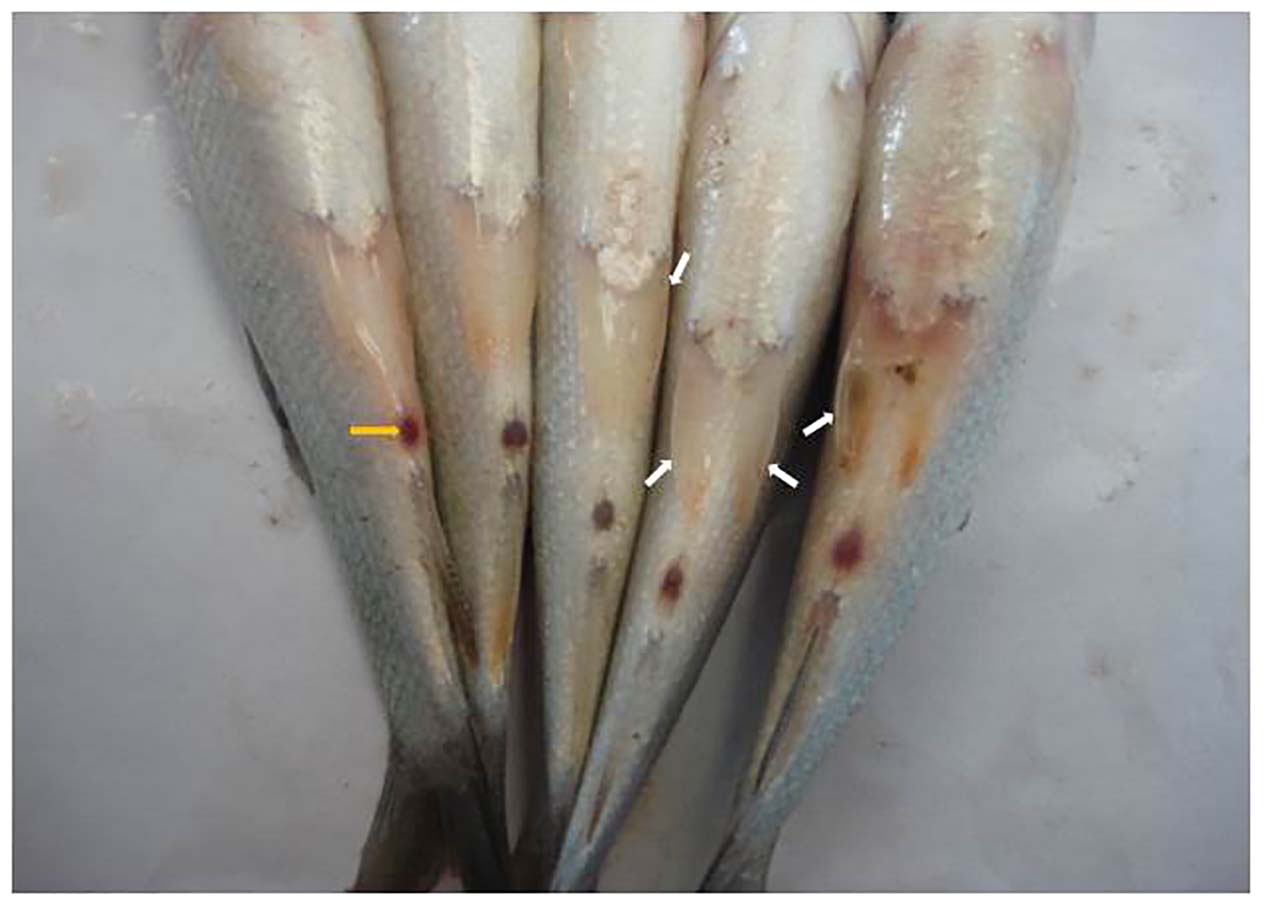
Fish injected with pathogenic bacteria *Aeromonas hydrophila* showing one or more typical signs of infection according to the stage of disease. Signs included hemorrhagia (large and irregular hemorrahages), shallow to deep necrotizing ulcers, and abdominal distension with sero-hemorrhagic fluids exuded from the inflamed vent. White arrows indicate edges of the ulcers. A yellow arrow indicates vent. Abdominal distension is clear in the right side fish. All fish shown are infected.

In field conditions, fishes are exposed to multiple stressors. To evaluate whether nonlethal exposure to low dose endosulfan aggravates pathogenic infectivity, fishes were exposed to a nonlethal dose of endosulfan for 37 days (pre-challenge) and subsequently injected intraperitoneally with the pathogenic bacteria *Aeromonas hydrophila* (post-challenge), and secondary stress responses were studied in fish fed with or unfed with lipotropic nutritional compounds. Successful experimental infection of *A. hydrophila* resulted in typical symptoms (**Figure 3**) such as hemorrhagia, shallow to deep necrotizing ulcers, and abdominal distension with sero-hemorrhagic fluids exuding from the vent. Dietary lipotropes potentiated the hematological profile (**Table 3**). Compared to control, RBC count was not affected by nonlethal exposure to endosulfan in pathogen-unchallenged fish. But in the same fish, lecithin supplementation was found to elevate (P<0.01) RBC count even under conditions of nonlethal endosulfan exposure. Following bacterial challenge, while the RBC count was significantly decreased (P<0.01) in endosulfan-exposed groups, there was no effect on WBC count. Choline, betaine and lecithin supplementation significantly (P<0.01) elevated the RBC and WBC count, which was even higher than control. The RBC and WBC count in post-challenge groups was higher than corresponding pre-challenge groups (P<0.05). Hemoglobin content was unaffected by treatments or bacterial challenge.

**Table 3:** Secondary stress response to low dose endosulfan-exposure in *L. rohita* fish fingerlings unfed or fed with lipotropes for 37 days: Pre-challange┼ and post-challenge^┼┼^ hematological profile.

Exposure/Diet	Control/Control	Endosulfan/Control	Endosulfan/Choline	Endosulfan/Betaine	Endosulfan/Lecithin	P-Value
RBC-Pre	1.66^ab^±0.08	1.35^a^±0.13	1.66^ab^±0.10	1.54^ab^±0.13	1.96^b^±0.18	0.001
RBC-Post	2.23^b^,**±0.03	2.08^a^, **±0.02	2.33^c^, **±0.03	2.41^d^, **±0.02	2.43^d^, ^*^±0.02	0.001
WBC-Pre	110^a^±1.15	105^a^±3.76	119^b^±1.33	129^c^±0.88	132^c^±2.03	0.001
WBC-Post	153^a^,**±3.46	148^a^,**±2.65	170^b^,**±1.67	181^c^,**±1.76	185^c^,**±0.58	0.001
Hb-pre	9.07±0.67	7.67±0.62	10.30±0.65	9.20±0.93	10.23±0.45	0.330
Hb-post	8.67±0.32	8.13±0.09	9.10±0.81	9.07±0.35	9.10±0.17	0.460

┼ Pre-challange blood samples were taken after 37 days of experiment. Subsequently, fish were challenged with the infectious bacteria, *A. hydrophila*, injected intraperitoneally and ^┼┼^post-challange samples were taken at the end of 7 days in surviving fish or just before sacrificing severely morbid fish.

Units: RBC count (x 10^6^ cells/mm^3^), WBC count (x 10^3^ cells/mm^3^) and Hemoglobin (Hb) g/dL.

**Indicates significant difference from pre-challenge values (P<0.01) with in a group by student's t-test.

a, b, c, d Means bearing different superscript letters in a row differ significantly against the P value indicated in the last column. Data expressed as Mean ± SE (n = 6).

Among serum proteins (**Table 4**), only albumin was significantly affected (P<0.05) by treatments, with no effects on serum total protein and globulin (both pre- and post-challenge). Compared to control, exposure to nonlethal levels of endosulfan had no effect on serum albumin, but albumin levels during the pre-challenge period were lower in betaine- and lecithin-supplemented (P<0.05) groups than in the control and endosulfan-exposed non-supplemented groups in the post-challenge state. Serum protein levels during post-challenge were lower than in corresponding pre-challenge groups (P<0.05).

**Table 4:** Secondary stress response to low dose endosulfan-exposure in *L. rohita* fish fingerlings unfed or fed with lipotropes for 37 days: Pre-challange┼ and post-challenge^┼┼^ serum protein profile.

Exposure/Diet	Control/Control	Endosulfan/Control	Endosulfan/Choline	Endosulfan/Betaine	Endosulfan/Lecithin	P-Value
TP-pre	8.45±0.30	8.40±1.58	8.66±1.42	9.06±0.68	9.25±1.22	0.97
TP-post	1.80*±0.08	1.54*±0.06	1.85*±0.06	1.84*±0.12	1.74*±0.12	0.38
Albumin-pre	1.77^bc^±0.05	1.91^c^±0.06	1.74^bc^±0.09	1.54^ab^±0.05	1.40^a^±0.11	0.006
Albumin-post	0.46^bc^, *±0.03	0.48^c^, *±0.01	0.34^ab^, *±0.01	0.31^a^,*±0.00	0.30^a^,*±0.08	0.02
Globulin-pre	6.68±0.26	6.49±1.63	6.93±1.48	7.52±0.73	7.85±1.33	0.91
Globulin-post	1.35*±0.10	1.06*±0.15	1.51*±0.16	1.53*±0.12	1.44*±0.09	0.24

┼ Pre-challange blood samples were taken after 37 days of experiment. Subsequently, fish were challenged with the infectious bacteria, *A. hydrophila*, injected intraperitoneally and ^┼┼^post-challange samples were taken at the end of 7 days in surviving fish or just before sacrificing severely morbid fish.

TP indicates Total Protein. Serum proteins expressed as g/dL.

*Indicates significant difference from pre-challenge value (P<0.01) with in a group by student's t-test.

a, b, c Means bearing different superscript letters in a row differ significantly against P value indicated in the last column. Data expressed as Mean ± SE (n = 6).

**Figure 4:**
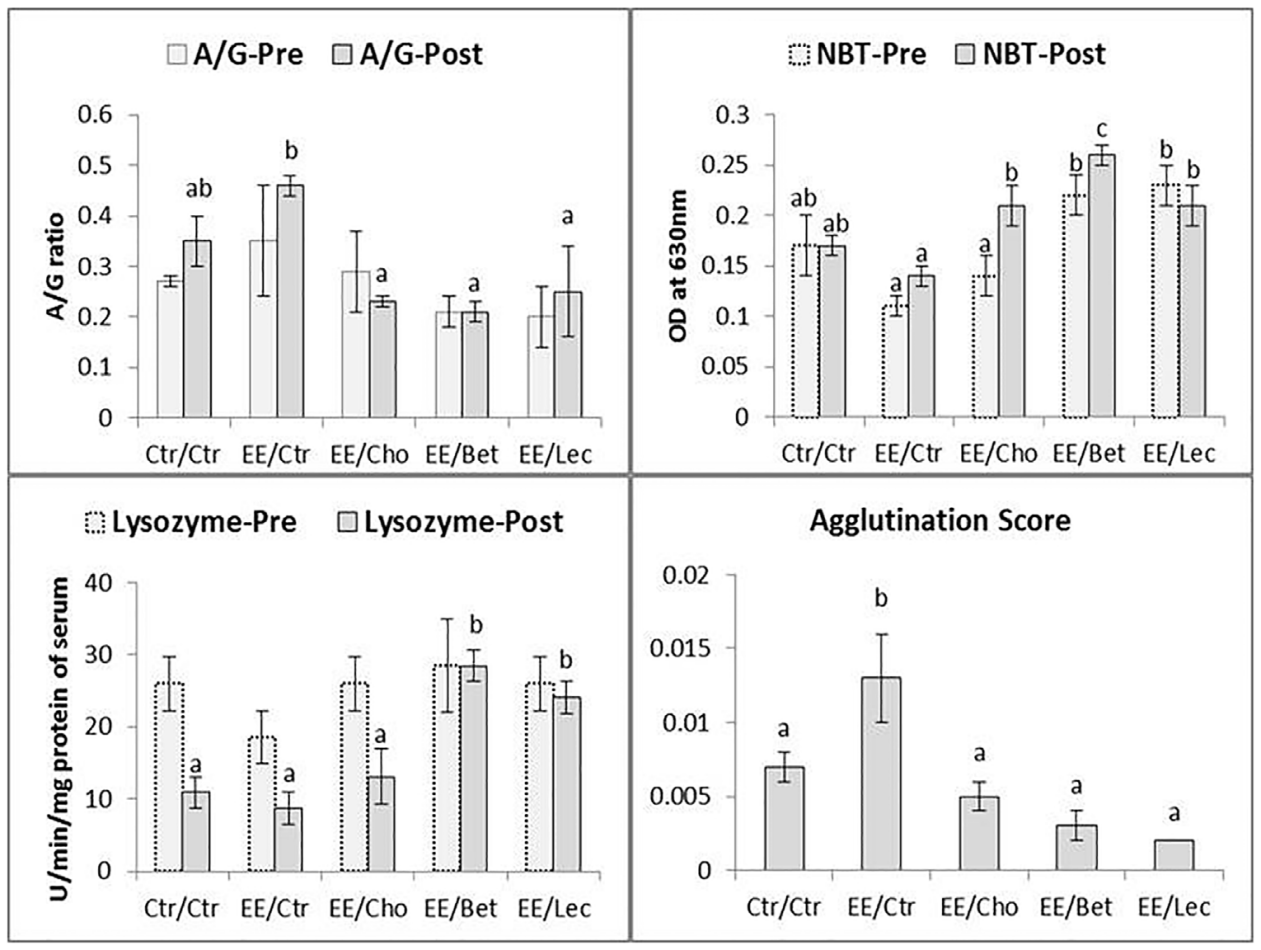
Secondary stress response to low dose endosulfan exposure in fish unfed or fed with lipotropes for 37 days: Pre- and post-challenge immunological responses. Pre-challenge samples were collected after 37 days of experiment. Fish were challenged with a pathogen, *A. hydrophila*, injected intraperitoneally and samples were collected at the end of 7 days post-challenge in surviving fish, or just before sacrificing severely morbid fish. Abbreviations for exposure/diet treatments of fish are the same as used in Figure 1: Ctr, control; EE, endosulfan exposed; Cho, choline; Bet, betaine; and Lec, lecithin. The values reported in bar charts represent the mean±SE. Bars bearing different letters (a, b, c) indicate significant differences between treatment means for respective attributes during a separate comparison of pre- or post-challenge data. Probability (P) values during pre-challenge: A: G ratio (P = 0.53), NBT (P = 0.013), lysozyme (P = 0.46). P values during post-challenge: A: G (P = 0.018), NBT (P = 0.002), lysozyme (P = 0.02) and agglutination score (P = 0.002). Number of observations (n): n = 6 for A: G, lysozyme and agglutination score, and n = 3 for NBT. Comparisons by t-test indicated significantly reduced (P<0.05) lysozyme post-challenge compared to pre-challenge within these respective groups: Ctr/Ctr, EE/Ctr and EE/Cho.

Lipotropes boosted the immunological response of fish exposed to pesticide and pathogenic stressors (**Figure 4**). Most significantly, nonlethal exposure to pesticide was found to have no effect on different nonspecific blood immune parameters such as the A: G ratio, lysozyme and NBT score during pre- or- post-challenge state. There were no differences between treatments for pre-challenge A: G ratio and lysozyme activity. None of the treatments were significantly different from control for post-challenge A: G ratios or lysozyme levels. Among the endosulfan-exposed but supplemented groups, betaine- and lecithin-fed groups exhibited higher lysozyme activity and the betaine-fed group exhibited a higher NBT score than in the control and endosulfan-exposed groups. The post-challenge bacterial agglutination titre was significantly enhanced (P<0.01) in endsosulfan-exposed groups. While the A: G ratio during post-challenge was not different from corresponding pre-challenge groups (P>0.05), post-challenge lysozyme in the control, endosulfan-exposed unsupplemented and choline-supplemented groups were lower and NBT in the choline-fed group was higher than in corresponding pre-challenge groups.

**Figure 5:**
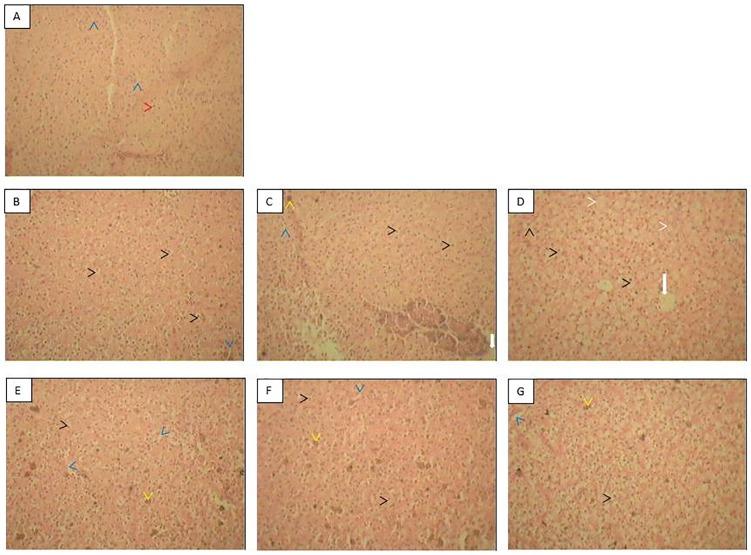
Secondary stress response to low dose endosulfan exposure in fish unfed or fed with lipotropes for 37 days: Histoarchitectural response. Histoarchitecture of the liver revealed a protective role for lipotropes in fish exposed to a nonlethal dose of endosulfan and intraperitoneally injected with the infectious bacteria, *A. hydrophila*. Pre-challenge samples were collected after 37 days of experiment. Post-challenge samples were collected at the end of 7 days in surviving fish or just before sacrificing severely morbid fish. Section from control fish (A), *A. hydrophila* injected fish (B), fish exposed to nonlethal dose of endosulfan (C) and also injected with *A. hydrophila* but either fed with no supplements (D), or fed with choline (E), betaine (F) or lecithin (G). Blue arrowhead: sinusoids, Red arrowhead: hepatocyte with nucleus, Black arrowhead: vacuole in hepatocyte, White arrow: central vein, White arrowhead: ghost cell without nucleus (due to karyolysis), Yellow arrowhead: focal inflammatory infiltrate. Histological changes are described in detail in the text.

Methyl donors provided histoarchitectural protection (**Figure 5**). Control liver histology showed normal polygonal hepatocytes with distinct nuclei and normal sinusoids (Figure 5A). In *A. hydrophila*-infected fish livers, slight degenerative changes with minimal hepatocellular hypertrophy and slight cytoplasmic vacuolation were observed (Figure 5B). In fish exposed only to a nonlethal dose of endosulfan, minimal vacuolation and hepatocellular hypertrophy were observed (Figure 5C). Exposure to a nonlethal dose of endosulfan aggravated *A. hydrophila*-induced pathological lesions in the livers of fish as evidenced by hepatocytocellular cloudy swelling/hypertrophy, more pronounced pyknotic/karyorrhectic nuclei, moderate cytoplasmic vacuolation and focal hepatocellular necrosis (Figure 5D). In the livers of fish exposed to a nonlethal dose of endosulfan and injected with *A. hydrophila* but fed with the methyl donors choline (Figure 5E), betaine (Figure 5F) or lecithin (Figure 5G), there were clear-cut signs of protection as revealed by reduced pathognomic histological lesions. Thus the protective efficacy of nutritional supplements on nonlethal low dose endosulfan-aggravated secondary stress profiles was evident.

### Low dose Endosulfan-exposed Fish Accumulate Residue, Have Enhanced Disease Susceptibility and Mortality (Tertiary Stress Responses) but Lipotropes Lower/Counteract It

**Figure 6:**
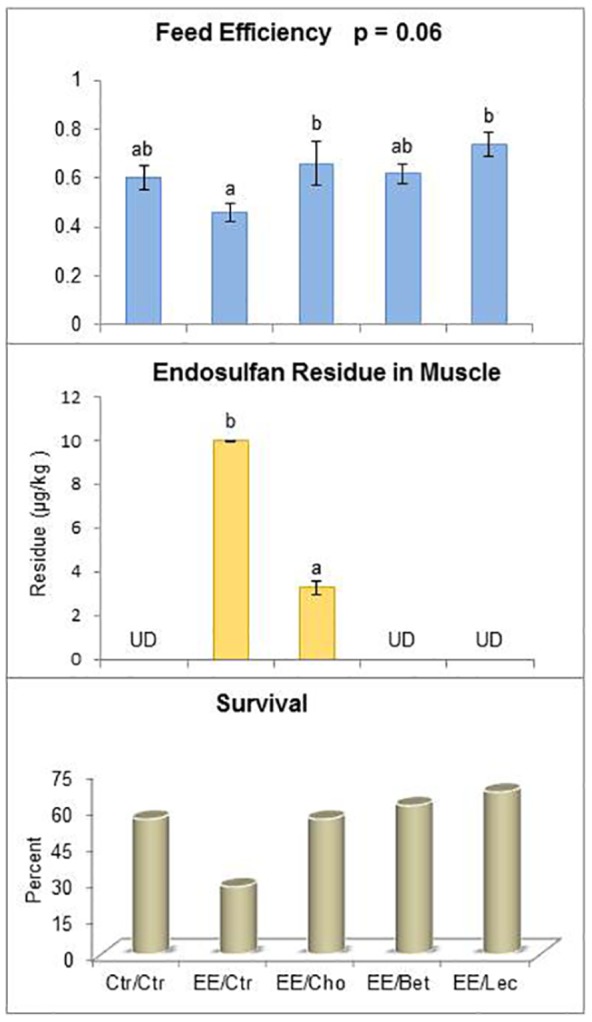
Tertiary stress response to low dose endosulfan exposure in fish unfed or fish fed with lipotropes for 37 days: Feed efficiency, residue accumulation and disease resistance against pathogen. Abbreviations for exposure/diet treatments of fish are the same as used in Figure 1: Ctr, control; EE, endosulfan-exposed; Cho, choline; Bet, betaine and Lec, lecithin. UD indicates undetectable. The values reported in bar charts represent the mean±SE. Bars bearing different letters (a, b) indicate significant differences between treatment means for that attribute. Probability (P) values: feed efficiency (P = 0.06), endosulfan residue in muscle (P≤0.01). Number of observations (n): feed efficiency data from 3 containers, n = 5 for endosulfan residue and n = 18 for survival. Fish were challenged with *A. hydrophila* after 37 days and post-challenge mortality/survival as a disease resistance indicator was recorded during a seven-day period.

Nonlethal exposure of fish to endosulfan had no effect on the initial and final body weights of the fish among different treatment groups (P>0.05) (**Table 5**), however, there was a slight variation in feed efficiency (weight gained per unit feed) (P = 0.06) (**Figure 6**). Importantly, the residue level in endosulfan-exposed but choline-fed fish was significantly reduced, while endosulfan was undetectable in other lipotrope-fed fish (P<0.01). The survival rate with nonlethal endosulfan exposure was 50 percent less (55.56% in control vs. 27.78% in endosulfan exposure). Choline supplementation prevented mortality as relative survival was similar to control, while survival in betaine- and lecithin-fed groups was 61.11 and 66.67 percent (i.e. 10% and 20% higher than control), respectively (Figure 6).

**Table 5:** Tertiary stress response to low dose endosulfan-exposure in *L. rohita* fish fingerlings unfed or fed with lipotropes for 37 days: Body weights.

Exposure/Diet	Control/Control	Endosulfan/Control	Endosulfan/Choline	Endosulfan/Betaine	Endosulfan/Lecithin	P-Value
Initial weight (g)	7.96±0.04	8.19±0.01	8.08±0.04	8.08±0.08	8.03±0.10	0.18
Final weight (g)	13.35±0.45	12.58±0.23	13.88±0.74	13.64±0.42	14.37±0.30	0.15

Data from three tubs expressed as Mean ± SE.

## Discussion

The study, in addition to providing greater ecophysiological insights into the already demonstrated [19], [28]–[30] synergistic effects of concurrent exposure to low dose pesticides and other stressors as a cause for species decline and reduced productivity, also prescribes practical preventive or mitigation strategies that would be useful to aquaculturists and biodiversity conservators. The integrated biological response to stressors is also a result of methylation of genes in stress-modulating LHPA circuitry (such as the neuropeptide corticotrophin-releasing factor [31], oxytocin and brain-derived neurotrophic factor [32], vitellogenin-1 [33] and the immune system [34]. This study under simulated conditions attempted to correct possible epigenetic dysregulation as noticed in other toxicities [7] through supplementation of methyl donor compounds like choline, betaine and lecithin with metabolic interrelationship. Lipotropes are used in synthesis of useful compounds such as creatine and phosphatidycholine (PC) [35] and for maintenance of epigenetic methylation. Our selection of the most appropriate compounds to protect fish from very low dose endosulfan-induced oxidative stress is also supported by recent ^1^H-NMR-based metabolic fingerprint report [36], which showed a decrease in choline content and lipid LDL in mice exposed to low doses of endosulfan.

The major sources of methyl groups for practical diets are betaine, choline, methionine and the choline derivative lecithin, which is a nutritionally superior source of choline. Aside from containing phospholipids like PC, phosphatidyl inositol, phosphatidyl ethanolamine and phosphatidic acid, soylecithin contains oil, some sterols and B group vitamins. About 40–50 percent of total phospholipids in eukaryotic cell membranes are PC. As even low dose endosulfan is known to damage cell membranes [37], lecithin may be involved in cellular homeostasis maintenance by keeping the cell membrane intact or providing components for repair. Betaine, being a compatible osmolyte, increases the water retention of cells, replaces inorganic salts, and protects intracellular enzymes against osmotically- or temperature-induced inactivation [38]. Choline is also an important component of some plasmalogens, sphingomyelins and lecithin and acts as a source of methyl groups, via betaine, for the synthesis of various methylated metabolites.

Physiological methods and concepts can be useful in conservation biology [39], and the link between diet quantity or quality [40] and the state of fasting and feeding and concentration of dietary macronutrients like dietary protein and lipid [41] and to some extent micronutrients has been studied, but the role of many other micronutrients in facilitating detoxification is not well delineated. Lipotropes have important functions in health and disease and the literature is centered around hepatotoxicity and carcinogenicity [42], [43]. As the liver is the principal organ for detoxification of endosulfan in fish [44] and pesticides also damage cellular membranes (composed of phospholipids), we used lipotropes for potentiating the detoxification capacity of fish. This is the first paper showing mechanistic effects of lipotrope compounds in ecotoxicity.

In a quest to regain homeostasis after low dose toxicity-induced physiological perturbations, the foremost stress response is a primary response consisting of catecholamine and glucocorticoid (cortisol) hormone, which is dependent on the duration and strength of the stressor [45]. Significantly elevated cortisol level was noted in the same fish species from the same stock (as used in the study) exposed to the nonlethal endosulfan from the same batch [25] at the end of a sixty day study. However, groups fed lipotropes on a preventive basis had these values significantly lower. Endosulfan has been known to damage the endocrine system and reproductive system [46] and among the negative effects of organochlorines includes lower plasma concentrations of gonadotropin, testosterone and 11-ketotestosterone (11-KT) [47]. In this study, a high amount of vitellogenin (Vtg) and decreased 11-KT suggests that nonlethal exposure to endosulfan is estrogenic in nature [48]. It is well known that Vtg cannot normally be detected in male fish. But this study employed soybean meal and fish meal as practical protein sources, and phytoestrogens (genestein, daidzein, coumestrol and equol) [49] from soybean meal and sex steroids (estradiol and estrone) from fish meal [50] are well known and potent Vtg inducers. Reported plasma levels of <10 ng/ml for male minnows, bream, gudgeon and zebrafish and 15 ng/ml for roach fish [51] agree with those found in this study. The fish used in our experiment were immature and possibly no sexual distinctions existed in the liver (where vitellogenin is synthesized) at this stage, as is also noted for other species [52]. The lack of significant differences in Vtg induction in males and females was similar to reports on immature fish (barfin plaice, *Liopsetta pinnifasciata*) from the moderately contaminated area of Amursky Bay in the Sea of Japan [53]. Abnormal Vtg induction in male summer flounder correlates with depression of plasma testosterone and with gonadal abnormalities [54]. The 11-Keto-testosterone in male *H. fossilis* was significantly decreased in response to hexachlorocyclohexane exposure [55]. It is likely that decreased sex steroids might be indicative of a change in the biosynthetic pathway of steroid hormones in the stressor group. The intervention might have been at the step where 17α-OH progesterone is converted into testosterone and then later to 11-keto testosterone. Hence, it would result in the depression of testosterone and 11-keto testosterone levels in the serum of males. However, low-dose endosulfan- exposed but choline-, betaine- and lecithin-fed groups had normal vitellogenin and 11-KT.

The primary hormonal response stimulates secondary responses, which are typically of short duration (up to hours) [56]; however, the stress response may persist during extended contaminant exposures [57]. We also studied secondary responses, such as changes in plasma and tissue metabolite levels, hematological features, and HSPs, which relate to physiological homeostasis [58].

Body organic matter, crude protein and ash and liver glycogen were not affected by a nonlethal level of endosulfan exposure for 37 days. In studies by Sarvanan et al. [59], glycogen content in the liver was decreased up to the 10th day, after that it gradually increased (p<0.05) from day 15 to day 25 of sublethal lindane (organochlorine) exposure. No change in body composition (or weight) in this study may indicate that the fish could satisfy their energy requirements and fish could accommodate stress due to nonlethal exposure to endosulfan. Endosulfan is a potent stimulator of the nervous system, and upon entering a fish's body it brings out several physiological alterations. Blood glucose levels increase upon exposure to organochlorine pesticides like endosulfan and lindane [25], [59]. In this study, the blood glucose levels (before challenge) were unchanged by treatments, but after challenge with pathogen blood glucose in the endosulfan-exposed group was increased, but was decreased in lipotrope-fed groups. Glucose levels in lipotrope-supplemented groups were on par with control, indicating efficient utilization of glucose from the blood, also attested by cortisol levels.

It is well established that exposure to organochlorine pesticides is associated with increased cytochrome P-450 (CYP) 1A1, a source of reactive oxygen species (ROS), as measured by EROD activity [60], [61]. Further, anti-oxidative enzyme (superoxide dismutase and catalase) activities, which protect cells against oxygen radical damage, and activities of phase II conjugating enzymes like GST (which helps in conjugation of products of phase I xenobiotic metabolism) also increase under pesticide stress [60], [61], as was also observed in this experiment and our earlier work [22]. Similarly, the activity of S-adenosyl methionine- (SAM) dependent methyl transferases (MTases) was also increased due to endosulfan exposure. The MTases use different substrates (e.g. DNA, RNA, protein, lipid and small molecules such as arsenic) and atoms for methylation (like C or S), and are involved in small biomolecule synthesis, elimination of small molecules and xenobiotics, stabilization of DNA, RNA and proteins, cellular signaling pathways, and protein synthesis. Most of the methylation reactions (about 85 percent) and 50 percent of all methionine metabolism take place in a single organ: the liver. Increased anxiety in female catechol-O-methyltransferase (COMT) knockout animals with increased cortisol levels and a role for COMT in modulating stress-related hormonal and immune parameters in a manner that depends on chronicity of the stressor has been demonstrated [62]. The liver is the principal organ that detoxifies endosulfan [63]. Lowered activity of antioxidative enzymes and phase II enzymes in xenobiotic metabolism in the treatment groups suggests that the supplementation of dietary methyl groups helps in detoxification in the liver. Improvement in antioxidative status with nutritional supplementation [64], even in sublethal exposure with pesticide or with stress [8], [41], has been reported.

Endosulfan generates reactive oxygen species (ROS) [65] and ROS-induced oxidative damage to mitochondria is a preliminary step to caspase-3 activation leading to apoptosis and necrosis [66]. The caspase-3 activity is induced by pesticides [67], and increased activity of this enzyme in liver and gill tissue of fishes exposed to nonlethal doses of endosulfan indicates its involvement in apoptosis. The HSPs affect cell survival by interacting with various components of the programmed cell death machinery, both upstream and downstream of the mitochondrial events [68]. Under stressed conditions, increased intracellular levels of HSP play an essential role in maintaining cellular homeostasis by assisting with the correct folding of nascent and stress-accumulated misfolded proteins, preventing protein aggregation or promoting selective degradation of misfolded or denatured proteins [69]. Induction of stress proteins is highly tissue-specific in animals [70]. Significantly higher (P<0.05) induction of HSP was observed in the gill and liver of the endosulfan-exposed group. Swimming Chinook salmon exposed for 30 days to sublethal levels of bleached kraft pulp mill effluent or sodium dodecylsulphate (100 percent survival in 100 percent effluent) showed significantly higher total HSP70 expression in the liver at all concentrations compared with the control group [71]. However, supplementation of choline, betaine and lecithin prevented induction of cellular HSP70 and caspase-3 in low dose endosulfan-exposed fish in this study.

The fish showed typical clinical signs of *A. hydrophila* infection and responded to the infection through changes in hematology, serum protein profile and immunology, as also noticed by Misra et al. [72]. While low dose endosulfan exposure showed no significant (P>0.05) negative effects on the majority of haemato-immunological parameters except decreased post-challenge RBC count and increased agglutination titer, significant (P<0.01) positive modulation of haemato-immunological profiles with dietary methyl donor compounds in *A. hydrophila*-challenged and endosulfan-exposed fish, as revealed by strong nonspecific or innate immune enhancement indicators such as enhanced WBC count [73], a lower A:G ratio [74], increased respiratory burst activity of phagocytes (measured by reduction of NBT by intracellular superoxide radicals produced by leucocytes) [75], increased lysosomal activity (except with choline) and restored agglutination titer [76]. A 60-day study from our laboratory utlizing *L. rohita* from the same source and the same endosulfan stock as this experiment observed differential effects of low dose endosulfan on varying immune parameters of the fish, which were ameliorated by dietary pyridoxine [25]. Fish are able to maintain their integrity through an innate immune system based on cell phagocytosis and secretion of soluble antimicrobial molecules. The innate system is characterized by being non-specific and therefore not dependent upon previous recognition of the surface structures of the invader. Immunostimulants can increase serum lysozyme activity, due to either an increase in the number of phagocytes secreting lysozyme, or to an increase in the amount of lysozyme synthesized per cell [77]. Elevation of lysozyme following immunostimulation has been demonstrated in a number of fish species [78]. Lysozyme has been found in mucus, serum and ova of fish [79]. Lysozyme may also act as an opsonin [80] and thereby help induce lysis of bacterial cell walls and stimulate the phagocytosis of bacteria and improve the innate immune response. A higher antibody agglutination titer was noticed in fingerlings inoculated with *Aeromonas hydrophila* and exposed to atrazine compared to inoculated nonexposed fish [81]. The erythropoiesis-stimulating effect of lipotropes noticed in this study corroborates with the observations of Rehulka and Minarik [82].

Protection of the histoarchitecture of the liver for effective detoxification and survival of fish exposed to multiple stressors is important. While the livers of fish exposed to a nonlethal dose of endosulfan had hypertrophied cells and very mild vacuolation and more vacuoles were noticed in the liver of fish that were only infected with *A. hydrophila*, the concurrent effect of both nonlethal exposure and bacterial infection was clearly additive in terms of hypertrophied cells, vacuolation and degenerative and necrotic changes. Vacuolation is caused by the protoxin of *A. hydrophila* which has been shown to be inserted into cell membranes, where its activation causes pore formation through a process of oligomerisation. This alters cell membrane K+ permeability, but the endoplasmic reticulum membranes are also altered, causing considerable ‘vacuolar’ distension [83]. Vacuolation in cells is generally seen as an adaptive physiological response for damage limitation, but very little is known about the intracellular homeostatic mechanisms which operate to restore the *status quo*. Where damage limitation fails, cells usually die quickly [84]. Provision of a good diet containing lipotropes thus appears to be a factor in fish attempts to maintain cellular homeostasis and histoarchitecure. While similar pathological lesions were observed in the liver of *A. hydrophila*-infected tilapia [85] and in Japanese flounder (*Paralichthys olivaceus*) caused by another infectious bacteria, *Edwardsiella tarda* [86], and independent reports also exist for similar liver pathology in fish exposed to sublethal endosulfan [87], there appears to be no literature that demonstrates the aggravating effect of low dose pesticide on liver lesions due to infectious bacteria or possible protective measures.

Choline, lecithin and betaine were found to have immunostimulatory role in fish in this study. The literature on the use of lipotropes in fish under nonlethal exposure in general and on their anti-oxidative and immunostimulatory properties in particular is scanty. We recently reported that these lipotropes promote immunobiochemical plasticity and protect fish against low-dose pesticide-induced oxidative stress during a 21-day experiment [88]. Unlike in earlier reports [88], in the present experiments the fish were challenged with pathogenic bacteria (*A. hydrophila*), a mandatory test to declare a compound to be an immunostimulant. Earlier, Klasing et al. [89] also demonstrated a modulatory effect of dietary betaine on the pathogenesis of *E. acervulina* infection in chicks and attributed the protective effect of betaine to enhancement of monocyte chemotaxis and nitrous oxide production by heterophils and macrophages. Methionine and betaine have shown immunomodulation in chicks [89], [90]. Their importance in immune responses may be due to their role in DNA-methylation occurring during immune recognition and antibody production [91]. In addition, the metabolic product of betaine, dimethylglycine, has been shown to enhance both the humoral and cell-mediated immune responses in humans [92] and in mice [93], although the mechanisms of the effect are unknown. Choline is a precursor of betaine, acetylcholine (neurotransmitter) and phosphatidylcholine (PC). Mustafa et al. [94] could not find immunostimulation with 0.25 percent phosphatidylcholine in Nile Tilapia, *Oreochromis niloticus*, reared at cold temperature. With around 20 percent PC in lecithin [95], the PC in this study (i.e. 0.4 percent) was higher than that of Mustafa et al. [94], in addition to other components provided by lecithin. Phospholipids are the key players in apoptosis and immune regulation [96] and the strong responses obtained indicate this fact.

Under extended exposure, as in this study, secondary stress responses may give rise to tertiary stress responses that will be detrimental to the organism's survival and reproduction [97]. Tertiary (organismal) reponses like initial and final body weight changes and feed efficiency showed non-significant differences among different treatment groups (P>0.05), which is in agreement with earlier reports on sublethal phenol [98] and sublethal dimethoate and malathion [99].

The primary stress response (endocrine hormones), secondary cellular stress responses such as activities of anti-oxidant enzymes (SOD and catalase), phase II (conjugation of xenobiotic metabolites) enzymes involved in detoxification (GST and methyltransferase), HSP70 and caspase, immune responses, disease susceptibility and survival and pathological histoarchitecture corresponded with the bioaccumulation of pesticide. The residue level in endosulfan-exposed but choline-fed fish was significantly reduced, while endosulfan was not detectable in betaine- and lecithin-fed fish (P<0.01). This is expected in a short duration study with low nonlethal dose of endosulfan as this using an otherwise balanced diet supplemented with lipotropes. An earlier report from our laboratory [8] indicated a potential role of nutritional intervention such as high protein (also means higher methionine, a methyl donor) and ascorbic acid in mitigating endosulfan toxicity through enhancing the liver's detoxification ability, leading to decreased residue accumulation in spotted murrel fish, *Channa punctatus*. Similarly, less toxicity but no mortality and less residue accumulation was reported when methionine was increased from 0.96 to 2.2 percent in the diet of rainbow trout subjected to dieldrin (an organochlorine) toxicity [100]. This is consistent with advances in the understanding of mechanisms that govern detoxification of foreign compounds, which revealed that diets (especially micronutrients) can have important impact on the efficacy of phase I and II enzymes [101]. The survival rate under a nonlethal dose decreased by 50 percent (55.56% for controls vs. 27.78% for endosulfan exposure). Choline supplementation prevented mortality as relative survival was similar to control, while survival in betaine and lecithin groups was 61.11 and 66.67 percent (i.e. 10% and 20% higher than control). Relyea and Mills [28] reported that predator-induced stress makes the pesticide carbaryl more deadly to gray treefrog tadpoles (*Hyla versicolor*) and is a reason for species decline. Increased susceptibility of baby salmon [19], and silver catfish fingerlings [22] to pathogens after exposure to harmful compounds at ecologically relevant levels and a decrease in the mean time to death after infection [23] along with the results of the current study sheds light on the synergistic effects of pesticides and pathogens on species disappearance. However, preventative measures as proposed can be used for conservation and resurrection of a declining population with a caveat that requirements of these nutrients for specific functions under specific conditions would be different.

## Conclusions

The comprehensive stress response in fish studied in this experiment realistically resonates with the state of aquatic animal health worldwide. Although, we used just one organo-chlorine pesticide at a nonlethal level, the severity of implications to health of concurrent exposure to a cocktail of low dose pesticides and biotic/abiotic stressors may be far greater, as can be correlated with the noticeable enormous fish biodiversity loss worldwide. We found synergistic effects of exposures to very low concentrations of a pesticide and pathogen infection, leading to further decreased immunocompetence and enhanced mortality in fish with already compromised stress responses, which can be counteracted with dietary lipotropic compounds that enhance immunity and the detoxification efficiency of the liver. This physiological basis indicates that nonlethal pesticide toxicities, along with induced nutritional deficiency stress, may be a driver for aquatic species decline or extinction, and appropriate strategies like dietary lipotropes may be used for resurrecting the endangered and declining aquatic species.

## Supporting Information

Table S1 **Physico-chemical parameters of water during the experimental period in different experimental groups.**
(DOC)
