# Supplementary material for: Lipotropes Protect against Pathogen-Aggravated Stress and Mortality in Low Dose Pesticide-Exposed Fish
Source: PLoS One. 2014 Apr 1;9(4):e93499. doi: 10.1371/journal.pone.0093499 (PMC3972094; doi:10.1371/journal.pone.0093499)
Supplement: Table S1 — Physico-chemical parameters of water during the experimental period in different experimental groups. (DOC) [file pone.0093499.s001.doc]

**Table S1.** Physico-chemical parameters of water during the experimental period for different experimental groups

|  | Exposure/ Diet | | | | |
| --- | --- | --- | --- | --- | --- |
| Parameter | Control / Control | Endosulfan/ Control | Endosulfan/ Choline | Endosulfan/ Betaine | Endosulfan/ Lecithin |
| Temperature(^0^C) | 26.6-28.5 | 26.5-28.7 | 26.4-28.8 | 26.5-28.4 | 26.4-28.6 |
| pH | 7.6-8.4 | 7.7-8.6 | 7.8-8.5 | 7.5-8.3 | 7.6-8.3 |
| DO_2_(mg/l) | 6.6-7.8 | 6.5-7.6 | 6.7-7.2 | 6.4-7.6 | 6.4-7.4 |
| Free CO_2_(mg/l) | ND | ND | ND | ND | ND |
| Hardness(mg/l) | 237-241 | 238-245 | 237-243 | 236-244 | 237-242 |
| Ammonia-N(mg/l) | 0.22-0.27 | 0.14-0.19 | 0.18-0.25 | 0.20-0.25 | 0.19-0.21 |
| Nitrite-N(mg/l) | 0.001-0.002 | 0.003-0.005 | 0.002-0.003 | 0.001-0.004 | 0.002-0.004 |
| Nitrate-N(mg/l) | 0.002-0.04 | 0.04-0.06 | 0.05-0.07 | 0.06-0.07 | 0.04-0.06 |
